# Supplementary material for: Structural Study of Metal Binding and Coordination in Ancient Metallo-β-Lactamase PNGM-1 Variants
Source: Int J Mol Sci. 2020 Jul 12;21(14):4926. doi: 10.3390/ijms21144926 (PMC7404133; doi:10.3390/ijms21144926)
Supplement: Supplementary file 1 [file ijms-21-04926-s001.pdf]

SUPPLEMENTARY INFORMATION

Structural study of metal binding and coordination of ancient metallo-β-lactamase PNGM-1 variants

Yoon Sik Park[1‡], Tae Yeong Kim[2‡], Hyunjae Park[1], Jun Hun Lee[2], Diem Quynh Nguyen[1], Sang Hee Lee[2¶] and Lin-Woo Kang[1¶]

[1]Department of Biological Sciences, Konkuk University, 120 Neungdong-ro, Gwangjin-gu, Seoul 05029, Republic of Korea; [2]National Leading Research Laboratory of Drug Resistance Proteomics, Department of Biological Sciences, Myongji University, 116 Myongjiro, Yongin, Gyeonggido 17058, Republic of Korea

Supplemental table

Table S1. The average distances (Å) between metal ion and coordination partner of eight protomers in the structure of PNGM-1 wild-type

	Zn1			Zn2	
	Average[a]	STDEV[b]		Average[a]	STDEV[b]
1	2.26	0.13	1'	2.26	0.05
2	2.15	0.08	2'	2.34	0.17
3	2.05	0.09	3'	2.18	0.12
4	2.19	0.12	4'	2.18	0.09
5	2.25	0.05	5'	2.35	0.13
6	2.99	0.14	6'	3.06	0.16
Total average[c]	2.31	0.10	Total average[c]	2.39	0.12

The label of metal coordination is same with Fig. S1.

[a]The distance average of the corresponding metal coordination in eight protomers.

[b]The distance standard deviation of the corresponding metal coordination in eight protomers.

[c]The average of six distances or standard deviations of the metal coordination values at Zn1 and Zn2.

Table S2. B factor values of metal ions and water molecules in the active site

	PNGM-1 WT		H91A		H93A	
	Average	STDEV	Average	STDEV	Average	STDEV
Zn1	19.88	2.65	N/A	N/A	N/A	N/A
Zn2	28.30	2.54	21.42	2.24	9.37	1.20
W^{cat}	24.81	4.12	19.25	5.38	10.61	1.27
W^{noncat}	30.71	4.12	43.80	15.49	26.80	6.50

	H96A		H257A		H279A	
	Average	STDEV	Average	STDEV	Average	STDEV
Zn1	23.77	3.11	18.85	1.41	17.41	6.82
Zn2	N/A	N/A	20.72	3.46	N/A	N/A
W^{cat}	N/A	N/A	14.95	3.06	17.50	9.45
W^{noncat}	N/A	N/A	37.83	2.31	25.11	12.88

Average, the average B factor of the corresponding atoms in protomers.

STDEV, the standard deviation of B factor of the corresponding atoms in protomers.

N/A, non-available

Table S3. Crystallization conditions of PNGM-1 wild-type and variants

PNGM-1 wild-type

10	%(w/v)	PEG 8000
0.1	M	Sodium acetate buffer pH 7.0
0.2	M	MgCl2
20	%(v/v)	glycerol

H96A

10	%(w/v)	PEG 3350
0.1	M	Sodium acetate buffer pH 5.4
0.15	M	Sodium formate
0.08	M	Calcium chloride

H91A

10	%(w/v)	PEG 3350
0.1	M	Sodium acetate buffer pH 5.4
0.15	M	Sodium formate
0.08	M	Calcium chloride

H257A

7.5	%(w/v)	PEG 8000
0.1	M	Sodium acetate buffer pH 4.6
0.2	M	MgCl2
20	%(v/v)	Glycerol

H93A

10	%(w/v)	PEG 4000
0.05	M	HEPES pH 7.1
0.15	M	Magnesium acetate
0.2	M	Ammonium acetate

H279A

10	%(w/v)	PEG 4000
0.05	M	HEPES pH 7.5
0.15	M	Magnesium acetate
0.2	M	Ammonium acetate

Each protein samples were incubated at 287K for 1 week. Small protein crystals were grown into full size using macro-seeding method with the same mother liquor solution.

Supplemental figures

Fig. S1

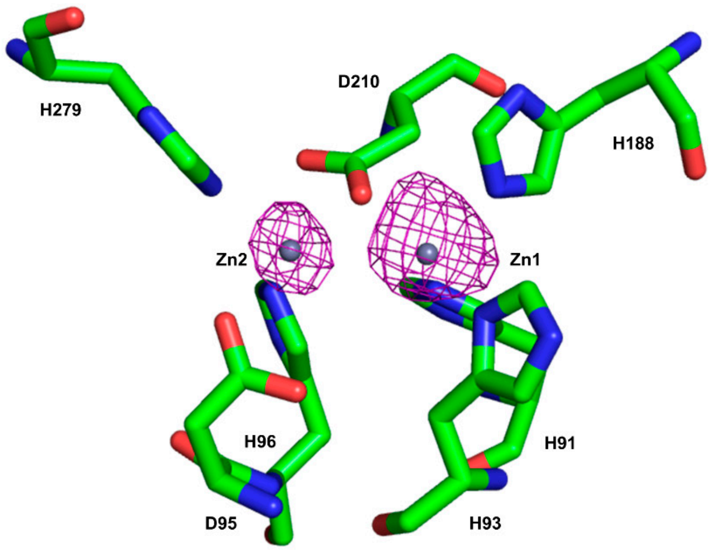

Fig. S1. The anomalous map of Zn ions in the active site of PNGM-1 wild-type. The anomalous map is contoured at 2.0 e/Å^3.

Fig. S2

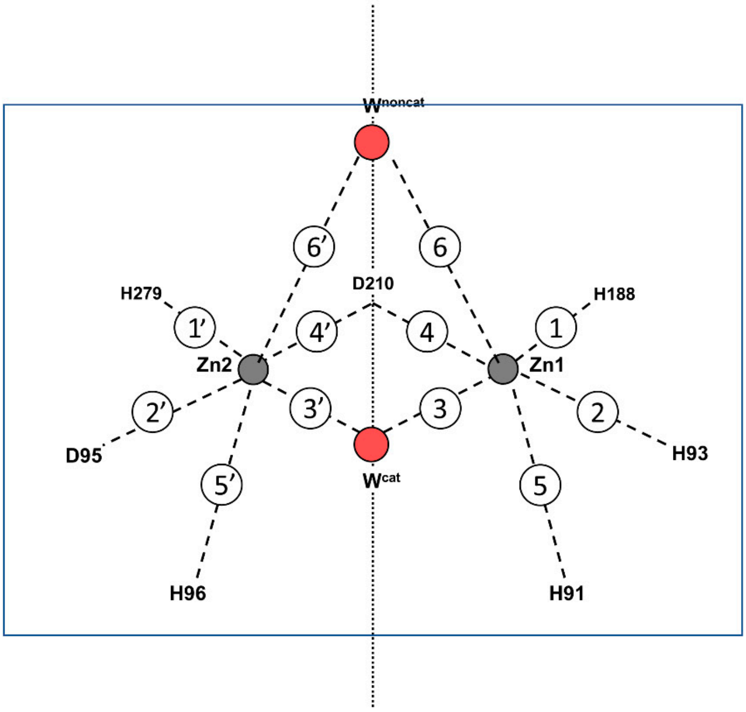

Fig. S2. The metal coordination geometry of two octahedrons in PNGM-1. Two octahedron structures are consequently combined to each other in a mirror-like symmetry. The label of metal coordination is same with Table S2.

Fig. S3

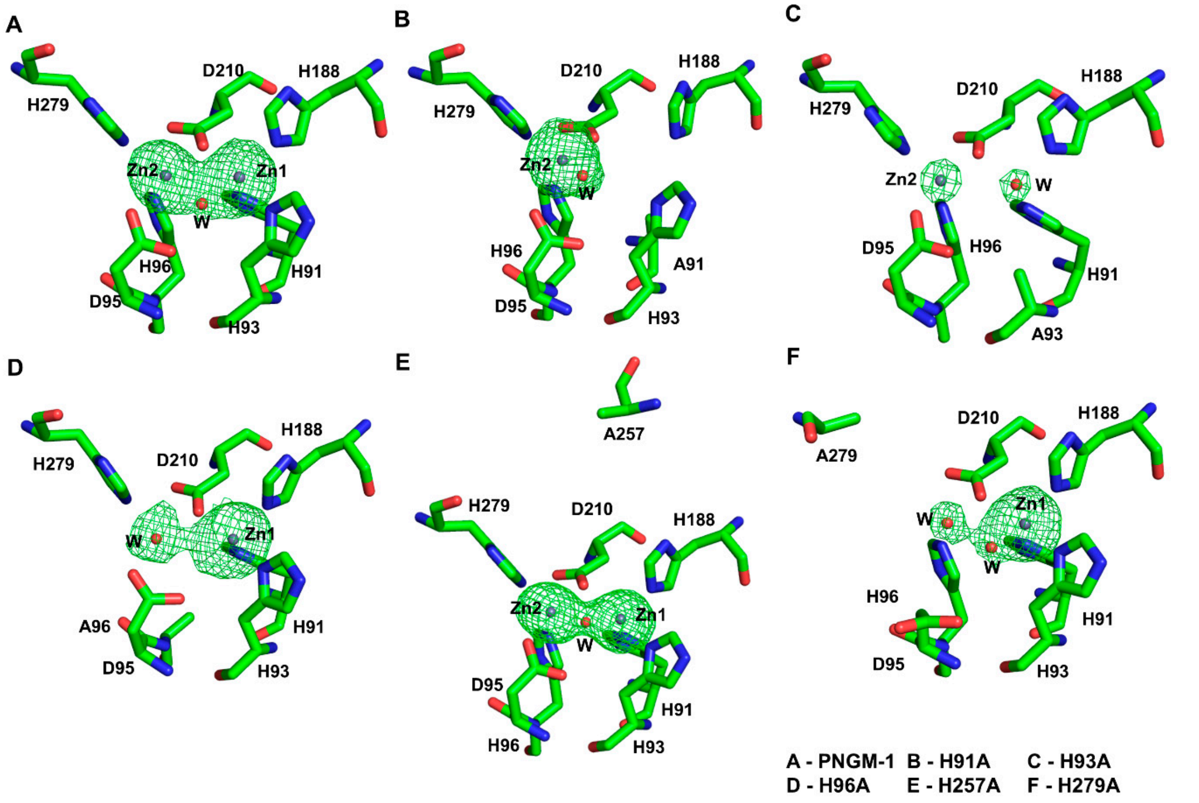

Fig. S3. The metal binding site of PNGM-1 wild-type and variants with the omit map: (a) PNGM-1 wild-type, (b) H91A variant, (c) H93A variant, (d) H96A variant, (e) H257A variant, and (f) H279A variant. The omit map of metal ions and water molecules is contoured at 4.0 e/Å^3 except the that of H93A variant (contoured at 10.0 e/Å^3).

Fig. S4

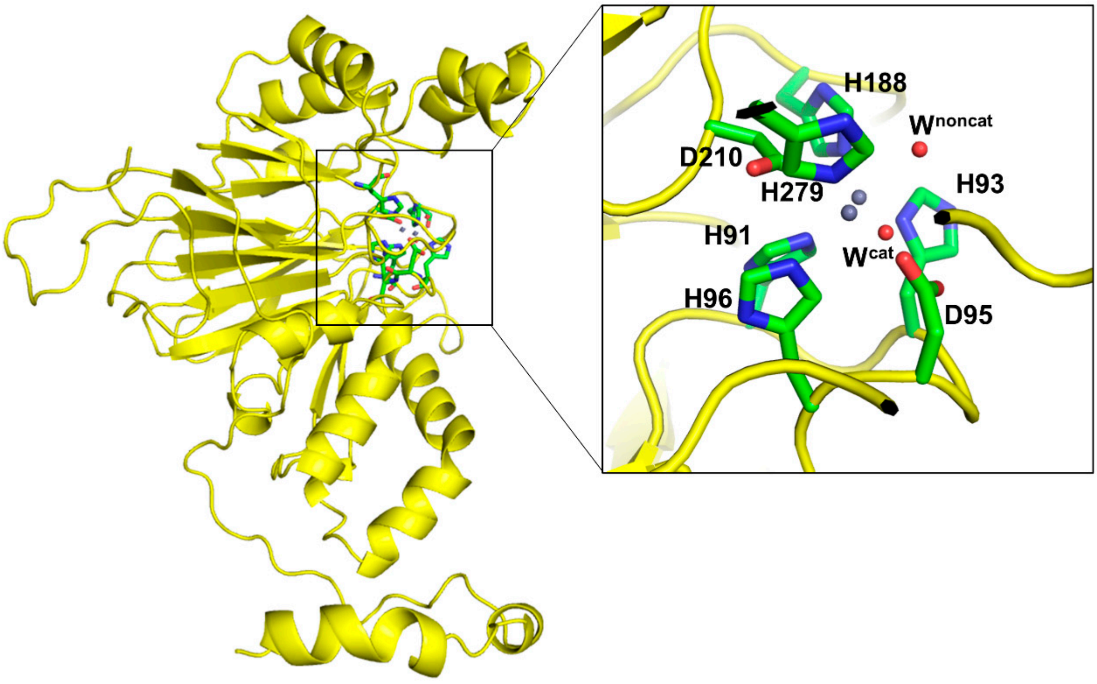

Fig. S4. The overall structure and metal binding site (inset view) of PNGM-1 wild-type. The metal ions (grey) and water molecules (red) are shown in balls and the metal coordinating residues (green) in sticks.

Fig. S5

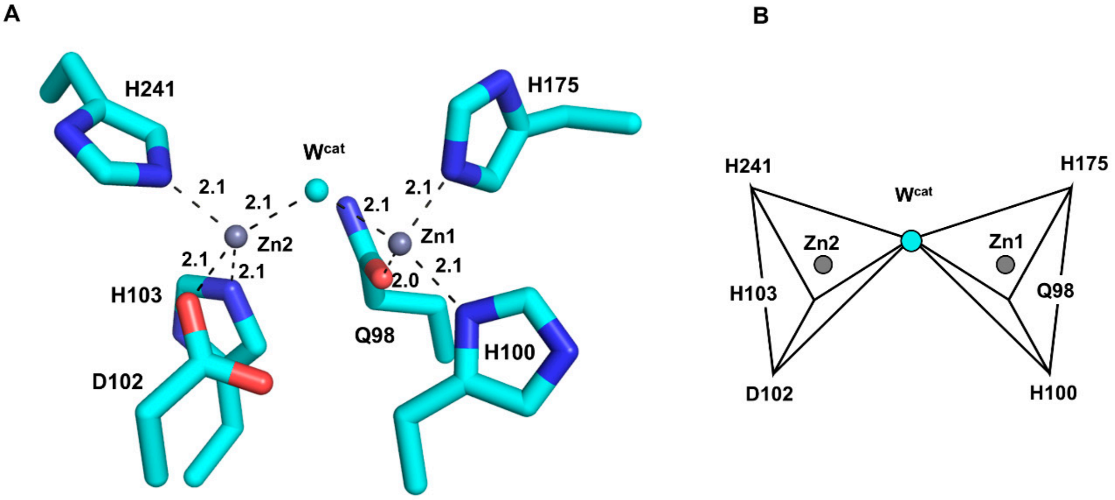

Fig. S5. The metal binding site (MBS) of M3 MBL GOB-18. (a) There are two MBS of Zn1 and Zn2. A zinc ion is bound at each MBS with four partners at the coordination distance of 2.0 ~ 2.1 Å. Grey balls represent a zinc ion and cyan ball, a water molecule. The coordination is shown in dashed lines. (b) Partners of the metal coordination have a tetrahedron shape. Two MBS consists of two consequently combined tetrahedrons having a zinc ion at the center.
